# Paraneoplastic myelitis associated with durvalumab treatment for extensive-stage small cell lung cancer

**DOI:** 10.1007/s10637-021-01154-x

**Published:** 2021-07-21

**Authors:** Lan Wang, Haiyan Lou, Bo Li, Jun Li, Yun-Mei Yang

**Affiliations:** 1 Department of Geriatrics, College of Medicine, The First Affiliated Hospital, Zhejiang University, Zhejiang Province, Hangzhou, 310003 China; 2 Department of Radiology, The First Affiliated Hospital, Zhejiang University School of Medicine, Zhejiang Province, 310003 Hangzhou, China; 3 Department of Pathology, The First Affiliated Hospital, Zhejiang University School of Medicine, Zhejiang Province, 310003 Hangzhou, China

**Keywords:** Durvalumab, Immune checkpoint inhibitor, Immune-related adverse effects, Paraneoplastic neurologic syndromes, Programmed cell death ligand protein 1, Small-cell Lung cancer

## Abstract

Paraneoplastic neurologic syndromes(PNSs) caused by immune checkpoint inhibitors(ICIs) is rare and requires clinicians to differentiate between disease progression and immune-related adverse effects(irAEs). We hereby report the case of immune-related myelitis accompanied by positive paraneoplastic autoantibodies following durvalumab treatment for extensive-stage small cell lung cancer (ES-SCLC). A 70-year-old Chinese woman with ES-SCLC was administered durvalumab with etoposid-platinum(EP) as first-line treatment. Four cycles after treatment with EP plus ICI, she developed immune-related myelitis with positive paraneoplastic autoantibodies (CV2, SOX1, ZIC4). Spinal MRI showed diffuse abnormal signal shadow in the cervicothoracic spinal cord. She was discontinued for chemotherapy, and treated with high-dose steroids, intravenous immunoglobulin and plasmapheresis, maintenance therapy with steroids resulted in a favorable neurologic outcome. This is the first report of durvalumab-related PNSs. We supposed that the development of paraneoplastic myelitis was causally related to immune activation by durvalumab. Prompt diagnosis and therapeutic intervention are essential for the effective treatment of paraneoplastic myelitis.

## Introduction

Small cell lung cancer (SCLC) is a deadly disease that represents about 15% of all lung cancers [1]. It is clinically characterized by a rapid growth and early metastatic widespread, around 70% of cases present with extensive-stage SCLC(ES-SCLC) at diagnosis [2]. The standard first-line chemotherapy for ES-SCLC has been etoposide combined with platinum (cisplatin or carboplatin). Although it has a high response rate, nearly all patients experienced quick disease relapse, with a median progression-free survival (PFS) of as long as 3 months, and poor survival outcomes, with a median overall survival (OS) of approximately 10 months [3].

Immune checkpoint inhibitors (ICIs) have significantly increased survival and they are becoming the standard of care in many malignancies [4]. The findings of a meta-analysis suggest that the combination of a PD-L1 inhibitor (durvalumab and atezolizumab) and etoposide based chemotherapy may be an optimal first-line treatment option for patients with ES-SCLC patients [5]. However, ICIs are known to cause unique immune-related adverse effects (irAEs). Common irAEs associated with the skin, gastro-intestinal (GI) tract, endocrine system, and liver are well documented with ICIs. Neurological irAEs occur in <1% of patients, especially immune-related myelitis. It may become irreversible and fatal in the absence of prompt and appropriate treatment [6].

Meanwhile, SCLC is most frequently associated with paraneoplastic neurologic syndromes (PNSs) [7]. PNSs can affect any part of the nervous system and results from an immune-mediated mechanism that produces direct damage to neural tissue rather than the direct invasion of a tumor or its metastasis [8]. PNSs are also rare, occurring in<1% of patients with cancer [9]. A recent review of PNSs that occurred following treatment with immune checkpoint inhibitors emphasized the clinical seriousness of these syndromes and the need for further investigation in the context of immunotherapy [10].

Here, we report a patient with ES-SCLC treated with first-line durvalumab combined with etoposide-platinum for 4 cycles and developed immune-related myelitis with positive paraneoplastic autoantibodies.

## Case presentation

**Fig. 1:**
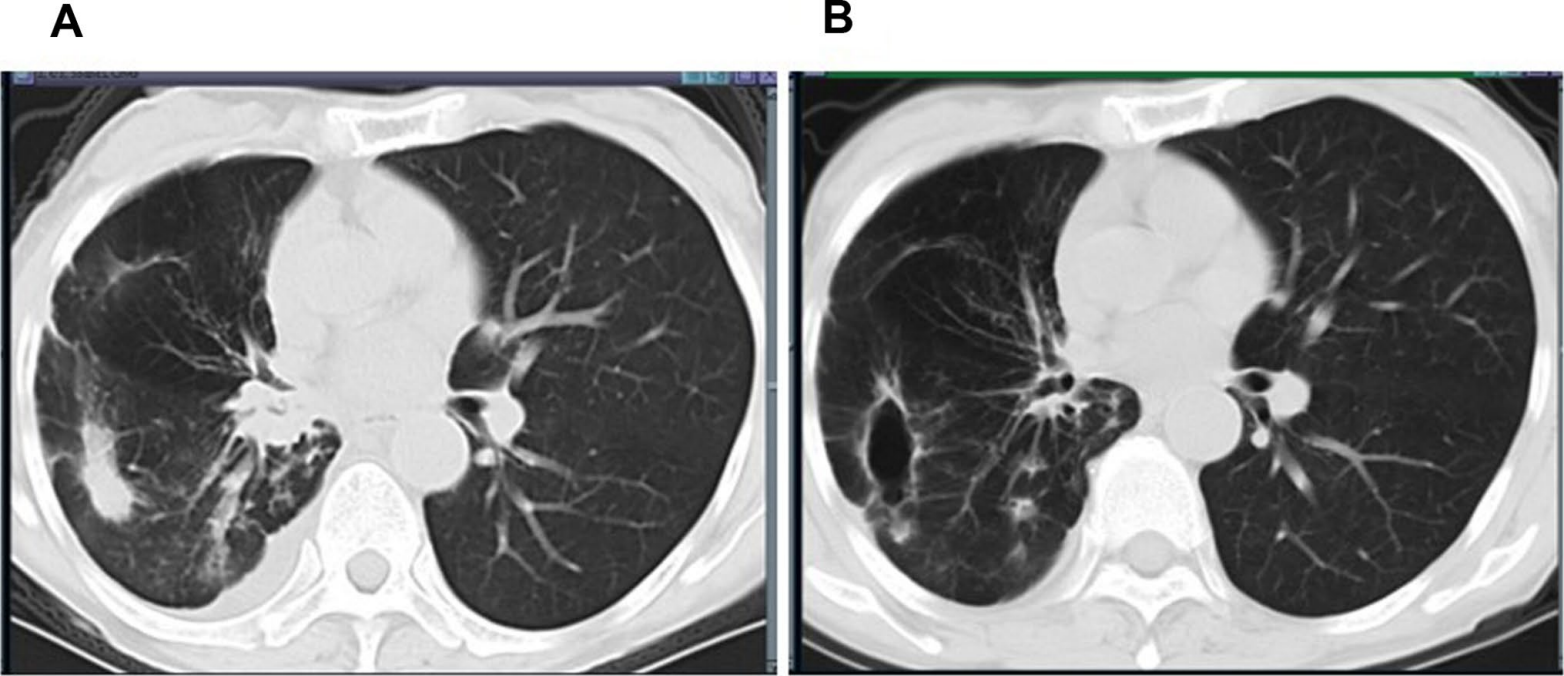
Chest CT scan showed an almost complete tumor response after three cycles of durvalumab treatment. **A** Before durvalumab treatment. CT plane scan revealed right lower lobe peripheral lung cancer with hilar lymph node metastasis. Simultaneously, CT contrast enhanced imges showed right lower pulmonary artery invasion, right lower pulmonary vein and right atrium tumor thrombus, right adrenal metastasis. **B** After durvalumab treatment for three cycles. CT lung window revealed Focal necrosis, thin-walled cavity formationthe hilar lymph nodes were significantly reduced. The morphology of hilum of lung returned to normal

**Fig. 2:**
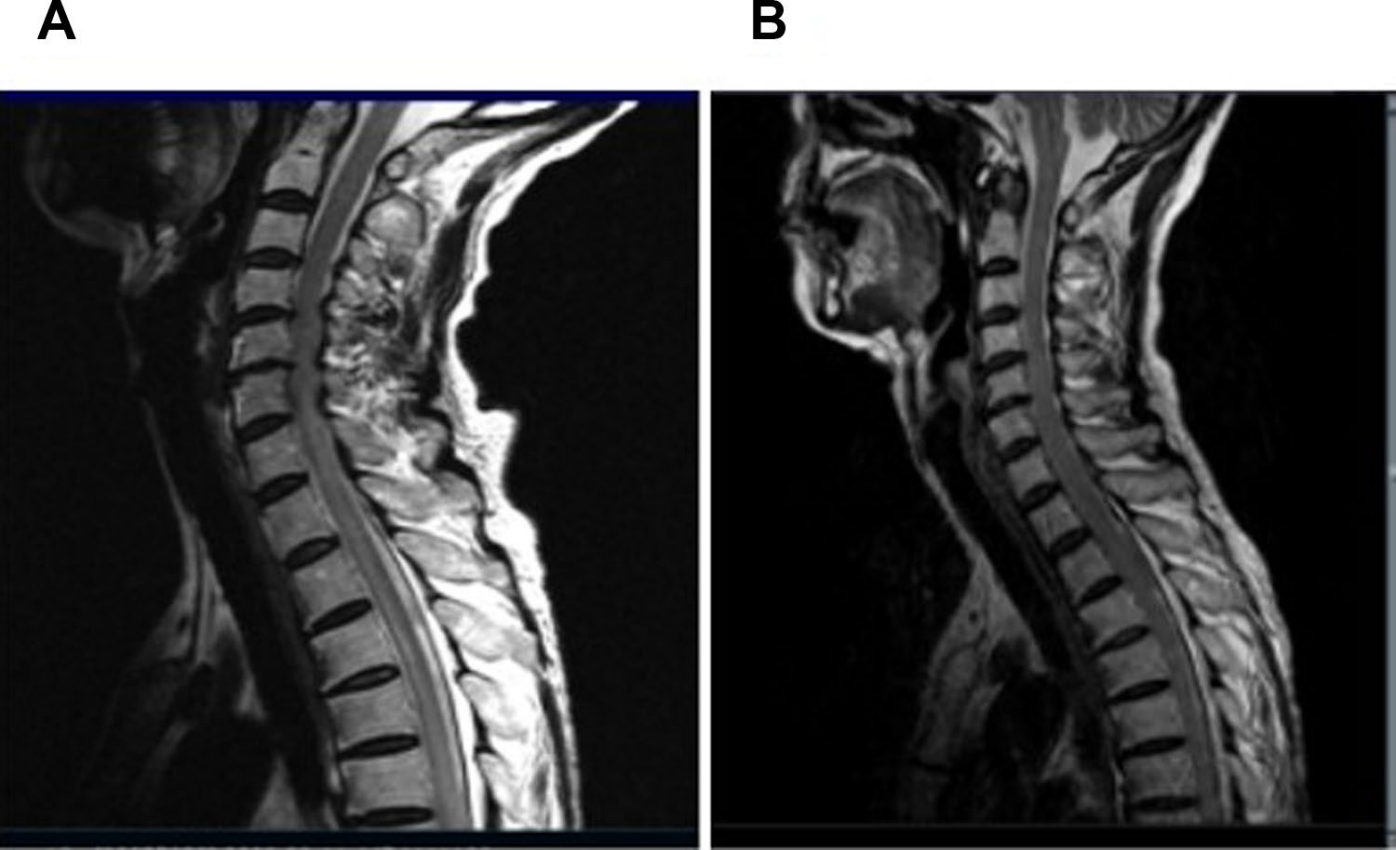
Immune injury of spinal cord was associated with durvalumab treatment. **A** Spinal magnetic resonance imaging (MRI) showed the swelling and hyperintensity of C7-T3 Segmentsspinal cord The lesions were laterally distributed in transverse section, and no enhancement was found in CE MRI. **B** Spinal MRI showed improvement after steroid pulse immunoglobulin and plasmapheresis therapy. The atrophy of the affected spinal cord were shown in the late images of follow-up

**Fig. 3:**
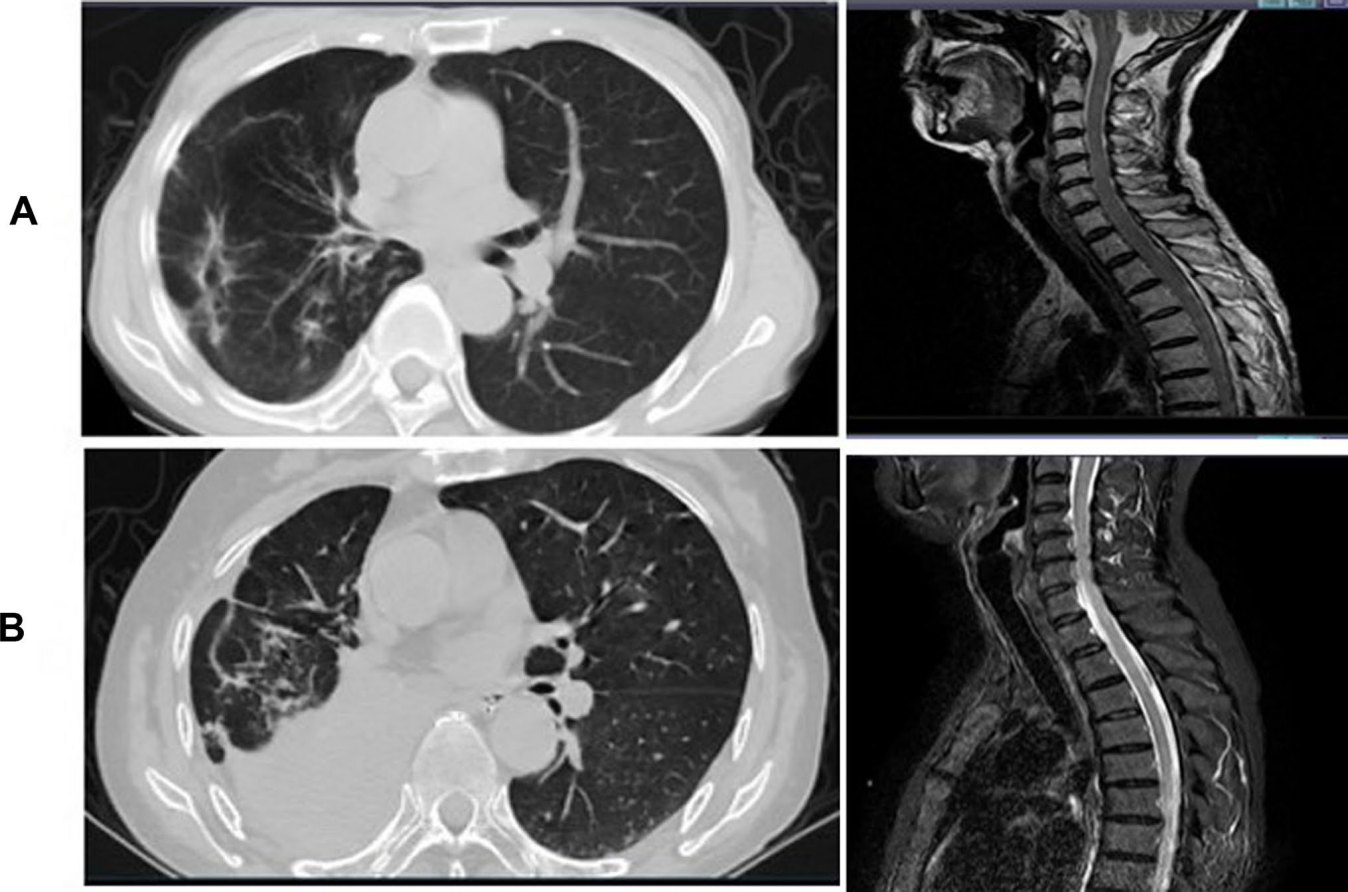
The progression of the lung tumor on chest CT were not synchronized with the changes of the cervicothoracic spinal cord on MRI. **A** After durvalumab treatment for three cycles. The right lower lobe lung cancer were obviously necrosis and formed thin-walled cavity formed, the hilar lymph nodes were significantly reduced. However, MRI of the spinal cord showed swellowed and ongitudinally extensive intramedullary hyperintensity in the cervicothoracic spinal cord. **B** The cancer progressed after cessation of anti-cancer therapy four months later. However, MRI showed atrophy of myelopathy.lesions of cervicothoracic spinal cord were stable

A 70-year-old Chinese women without any previous neurological illnesses was diagnosed with small cell lung cancer with synchronous adrenal metastases. The gene detection indicated that PD-1 was positive. She underwent four cycles of durvalumab plus etoposid-platinum chemotherapy as first-line chemotherapy. After 3 cycles therapy, computed tomography (CT) evaluation showed an almost complete tumor response (Fig. 1). However, ten days after the fourth chemotherapy, the patient developed numbness and weakness of the lower limbs, and gradually developed to the proximal thigh root and hands. She developed bladder dysfunction and was unable to walk unaided. She underwent a further neuro-oncologic evaluation. Spinal MRI showed diffuse abnormal signal shadow in the cervicothoracic spinal cord (Fig. 2). Brain MRI revealed no changes in the known brain metastases and no evidence of ischemic or hemorrhagic events. Central nerve demyelinating antibody test: anti MOG antibody was positive (titer 1:10), anti AQP4 and anti MBP antibody were negative. Paraneoplastic autoantibodies (CV2, SOX1, ZIC4) were positive as well. Analysis of the cerebrospinal fluid (CSF) showed normal white cell count, protein concentration of 123mg/dL, and normal glucose concentration of 2.7mmol/L(blood glucose 3.5mmol). CSF cytology was negative, and CSF cultures for bacteria, mycobacterium, and fungi were also negative. CSF tumor markers (carcinoembryonic antigen, squamous cell carcinoma) were all negative. She was diagnosed with immune-related myelitis. She was discontinued with chemotherapy and treated with high dose of steroids, intravenous immunoglobulin and plasmapheresis, maintenance therapy with small dose of steroids resulted in a favorable neurologic outcome. The cancer progressed after cessation of anti-cancer therapy four months later. However, her neurologic symptoms did not worsen. MRI showed that the morphology and signal of myelopathy were improved (Fig. 3). She underwent irinotecan-carboplatin chemotherapy for 3 cycles and her lesion in the right lower lobe were reduced. However, she did not complete fourth cycle of the chemotherapy due to grade 3 diarrhoea according to the Common Terminology Criteria for Adverse Events version 4.1. Therefore, she was treated with radiotherapy. Now she had achieved PR according to results of imaging assessment.

## Discussion

Immune checkpoint inhibitors (ICIs) is associated with a range of neurological immune-related adverse effects, including myasthenia gravis (MG), infammatory myositis, encephalitis/myelitis, meningitis, or Guillain–Barré syndrome (GBS) [11]. Most of the adverse events are occurring after weeks or months of ICI administration, some can occur very early after only one dose [12]. Moreover, SCLC is the malignancy that is most frequently associated with PNSs [13]. This patient developed neurological toxicity ten days after the fourth chemotherapy. However, her paraneoplastic autoantibodies (CV2, SOX1, ZIC4) were positive. Therefore, we face differential diagnosis between paraneoplastic neurologic syndrome and ICI neurological irAEs.

Recent studies suggested that ICIs can trigger both antibody-mediated and T-cell mediated paraneoplastic neurologic syndromes [14]. Another study indicated that sixteen patients had a newly diagnosed PNS after they received ICIs therapy. The median (range) time between immunotherapy initiation and the new diagnosis of a PNS was 1.6 months. Six (38%) patients had an objective tumor response at the time when the PNS appeared. Half of patients with a pre-existing PNS experienced a worsening of the corresponding symptoms after the initiation of anti-PD-1 or anti-PD-L1 immunotherapy [15]. In this case, the patient developed acute myelitis with anti-CV2 positive after she received durvalumab therapy. The time between immunotherapy initiation and neurological toxicity was 4.3 months. In a study of 23 patients anti-CV2 positive, 60% had a paraneoplastic cerebellar degeneration and 60% had a SCLC. The neurological symptoms precede the diagnosis of the tumour in 78% of the cases [16]. Another study showed that lung cancer cases had anti-Hu, CV2 or amphiphysin antibodies with non-classical clinical presentations should not be neglected for cancer associated PNS [17]. However, this patient did not have any previous neurological illnesses and clinical presentations before she received durvalumab therapy. The relationship between durvalumab-initiation, acute neurologic syndromes, and clinical improvement following durvalumab cessation and immunotherapy supports a cause-effect relationship. Therefore, we considered the neurological irAEs caused by durvalumab initiating PNSs. We should measure paraneoplastic autoantibodies of this patient before ICIs therapy.

In a series of 468 patients with and without PNS, 61 were ZIC-4 positive, including 92% of SCLC [18]. SOX1 antibodies are found in 64% of patients with Lambert-Eaton Myasthenic Syndrome(LEMS) in association with voltage-gated calcium channel antibodies as serological markers of SCLC [19]. They are also identified in 40% of patient with SCLC without neurological syndrome [19]. This patient was both ZIV-4 and SOX1 positive after receiving ICIs therapy, indicating neurological irAEs caused by durvalumab therapy were associated with PNS.. Although paraneoplastic myelitis may be a life-threatening complication of immunotherapy, this patient had an objective tumor response at the time when the PNS appeared. Moreover, the cancer progressed after cessation of anti-cancer therapy four months later. However, her neurologic symptoms did not worsen. MRI showed that the morphology and signal of myelopathy were improved. Hence, we thought that the enhanced immune response against the tumour can also target the nervous system. The immune-related myelitis was probably triggered by immunotherapy and not by tumor progression. A descriptive study showed that the exacerbation or appearance of a PNS can be associated with an effective tumor response soon after the initiation of immunotherapy [20]. The results were as same as our results. The progression-free survival (PFS) of the patient was 4 months. Moreover, the imaging findings showed that the progression of the lung tumor on chest CT were not synchronized with the changes of the cervicothoracic spinal cord on MRI. Therefore, we supposed that the development of paraneoplastic myelitis was causally related to immune activation by durvalumab.

For severe neurotoxicity (grade 3 or higher according to the Common Terminology Criteria for Adverse Events of the National Cancer Institute), current guidelines suggest management in the following order: ICI cessation, high-dose steroids, other T cell suppressive medications and intravenous immunoglobulin (IVIG) or plasmapheresis [21, 22]. In this case report, administration of high dose of steroids, intravenous immunoglobulin and plasmapheresis produced both clinical and imaging improvement.

## Conclusion

In conclusion, we present a case with PNSs affecting the spinal cord in a ES-SCLC patient treated with durvalumab combined with etoposide-platinum chemotherapy. This is the first report of durvalumab-related PNSs. We should measure paraneoplastic autoantibodies of SCLC patients before ICIs therapy and determine whether the use of ICIs in patients with serum onconeural antibodies but without neurological symptoms in order to decrease the risk of neurological irAEs. Differentiation between disease progression and side effects may be difficult and treatment decisions should be discussed in a multidisciplinary team. Immune checkpoint inhibitors should be discontinued and treatment with corticosteroids should be initiated early as the drug of first choice. Therapy may be escalated by intravenous immunoglobulin and plasmapheresis.

## Data Availability

The data in the current study are not publicly available to protect patient privacy, but the data in favor of these findings are available from the corresponding author if any reasonable request.

## References

[1] Wang BC, Xiao BY, Li PC, Kuang BH, Chen WB, Li PD, Lin GH, Liu Q (2020). Efficacy and safety of first-Line immunotherapy in combination with chemotherapy for patients with extensive-stage small cell lung cancer: A Systematic Review and Network Meta-Analysis. J Oncol.

[2] Facchinetti F, Di Maio M, Tiseo M (2020). Adding PD-1/PD-L1 Inhibitors to chemotherapy for the first-line treatment of extensive stage small cell lung cancer (SCLC): A meta-analysis of randomized trials. Cancers (Basel).

[3] Pujol JL, Greillier L, Audigier-Valette C, Moro-Sibilot D, Uwer L, Hureaux J, Guisier F, Carmier D, Madelaine J, Otto J, Gounant V, Merle P, Mourlanette P, Molinier O, Renault A, Rabeau A, Antoine M, Denis MG, Bommart S, Langlais A, Morin F, Souquet PJ (2019). A Randomized Non-Comparative Phase II Study of Anti-programmed cell death-ligand 1 atezolizumab or chemotherapy as second-line therapy in patients with small cell lung cancer: Results from the IFCT-1603 Trial. J Thorac Oncol.

[4] Ferrara R, Imbimbo M, Malouf R, Paget-Bailly S, Calais F, Marchal C, Westeel V (2020) Single or combined immune checkpoint inhibitors compared to first-line platinum-based chemotherapy with or without bevacizumab for people with advanced non-small cell lung cancer. Cochrane Database Syst Rev 12:CD013257 10.1002/14651858.CD013257.pub2 PMC8094159 33316104

[5] Zhou T, Zhang Z, Luo F, Zhao Y, Hou X, Liu T, Wang K, Zhao H, Huang Y, Zhang L (2020). Comparison of first-Line treatments for patients with extensive-stage small cell lung cancer: A systematic review and network meta-analysis. JAMA Netw Open 3(10):e2015748 10.1001/jamanetworkopen.2020.15748 PMC7573680 33074323

[6] Fellner A, Makranz C, Lotem M, Bokstein F, Taliansky A, Rosenberg S, Blumenthal DT, Mandel J, Fichman S, Kogan E, Steiner I, Siegal T, Lossos A, Yust-Katz S (2018). Neurologic complications of immune checkpoint inhibitors. J Neurooncol.

[7] Bentea G, Sculier C, Grigoriu B, Meert AP, Durieux V, Berghmans T, Sculier JP (2017). Autoimmune paraneoplastic syndromes associated to lung cancer: A systematic review of the literature: Part 3: Neurological paraneoplastic syndromes, involving the central nervous system. Lung Cancer.

[8] Vogrig A, Fouret M, Joubert B, Picard G, Rogemond V, Pinto AL, Muñiz-Castrillo S, Roger M, Raimbourg J, Dayen C, Grignou L, Pallix-Guyot M, Lannoy J, Ducray F, Desestret V, Psimaras D, Honnorat J (2019) Increased frequency of anti-Ma2 encephalitis associated with immune checkpoint inhibitors. Neurol Neuroimmunol Neuroinflamm 6(6):e604 10.1212/NXI.0000000000000604 PMC6705619 31454760

[9] Paz-Ares L, Dvorkin M, Chen Y, Reinmuth N, Hotta K, Trukhin D, Statsenko G, Hochmair MJ, Özgüroğlu M, Ji JH, Voitko O, Poltoratskiy A, Ponce S, Verderame F, Havel L, Bondarenko I, Kazarnowicz A, Losonczy G, Conev NV, Armstrong J, Byrne N, Shire N, Jiang H, Goldman JW (2019) CASPIAN investigators. Durvalumab plus platinum-etoposide versus platinum-etoposide in first-line treatment of extensive-stage small-cell lung cancer (CASPIAN): a randomised, controlled, open-label, phase 3 trial. Lancet 394(10212):1929–1939 10.1016/S0140-6736(19)32222-6 31590988

[10] Valencia-Sanchez C, Zekeridou A (2021) Paraneoplastic Neurological syndromes and beyond emerging with the introduction of immune checkpoint inhibitor cancer immunotherapy. Front Neurol 12:642800 10.3389/fneur.2021.642800 PMC8062756 33897597

[11] Pan PC, Haggiagi A (2019). Neurologic immune-related adverse events Associated with immune checkpoint inhibition. Curr Oncol Rep.

[12] Graus F, Dalmau J (2019). Paraneoplastic neurological syndromes in the era of immunecheckpoint inhibitors. Nat. Rev. Clin. Oncol.

[13] Horn L, Mansfield AS, Szczęsna A, Havel L, Krzakowski M, Hochmair MJ, Huemer F, Losonczy G, Johnson ML, Nishio M, Reck M, Mok T, Lam S, Shames DS, Liu J, Ding B, Lopez-Chavez A, Kabbinavar F, Lin W, Sandler A, Liu SV (2018) IMpower133 Study Group. First-line atezolizumab plus chemotherapy in extensive-stage small-cell lung cancer. N Engl J Med 379(23):2220-2229 10.1056/NEJMoa1809064 30280641

[14] Rogemond V, Honnorat J (2000) Anti-CV2 autoantibodies and paraneoplastic neurological syndromes. Clin Rev Allergy Immunol 19 (1): 51–59 10.1385/CRIAI:19:1:51 11064826

[15] Gill A, Perez MA, Perrone CM, Bae CJ, Pruitt AA, Lancaster E (2019) A case series of PD-1 inhibitor-associated paraneoplastic neurologic syndromes. J Neuroimmunol 334:576980 10.1016/j.jneuroim.2019.576980 31195181

[16] Gure O, Stockert E, Scanlan MJ, Keresztes RS, Jager D, Altorki NK, Old LJ, Chen YT (2000). Serological identifification of embryonic neural proteins as highly immunogenic tumor antigens in small cell lung cancer. Proc. Natl. Acad. Sci. U. S. A..

[17] Berger B, Bischler P, Dersch R, Hottenrott T, Rauer S, Stich O (2015). Non-classical paraneoplastic neurological syndromes associated with well-characterized antineuronal antibodies as compared to classical syndromes – more frequent than expected. J. Neurol. Sc.

[18] Sabater L, Bataller L, Suarez-Calvet M, Saiz A, Dalmau J, Graus F (2008). ZIC antibodies in paraneoplastic cerebellar degeneration and small cell lung cancer. J. Neuroimmunol.

[19] Sun X, Tan J, Sun H, Liu Y, Guan W, Jia J, Wang Z (2020). Anti-SOX1 antibodies in paraneoplastic neurological syndrome. Clin Neurol.

[20] Sabater L, Höftberger R, Boronat A, Saiz A, Dalmau J, Graus F (2013) Antibody repertoire in paraneoplastic cerebellar degeneration and small cell lung cancer. PLoS One 8(3):e60438 10.1371/journal.pone.0060438 PMC3607586 23536908

[21] Chen J, Wang J, Xu H (2021). Comparison of atezolizumab, durvalumab, pembrolizumab, and nivolumab as first-line treatment in patients with extensive-stage small cell lung cancer: A systematic review and network meta-analysis. Medicine (Baltimore)100(15):e25180 10.1097/MD.0000000000025180 PMC8051984 33847617

[22] Kim A, Keam B, Cheun H, Lee ST, Gook HS, Han MK (2019). Immune-checkpoint-inhibitor-induced severe autoimmune encephalitis treated by steroid and intravenous immunoglobulin. J Clin Neurol.

